# Trend prediction and influencing factors of the production comparative advantage of China’s main apple-producing provinces

**DOI:** 10.1371/journal.pone.0311912

**Published:** 2024-10-18

**Authors:** Wenping Ning, Fuhong Zhang, Meiling Zhang

**Affiliations:** School of Economics and Management, Shandong Agricultural University, Taian, Shandong, China; University of Southampton, MALAYSIA

## Abstract

**Introduction:**

The apple industry is an essential industry to assist in rural revitalization. However, in recent years, the urbanization, industrialization, globalization and climate change have brought various challenges to the apple industry in China’s main apple-producing provinces. Given this, effectively identifying, enhancing on apple production comparative advantage (APCA) is imperative to safeguard the long-term sustainable development of China’s apple industry. This study aims to explore the evolutionary trends and influencing factors of APCA, and to provide quantitative support for the formulation of scientific and effective apple production policies.

**Methods:**

In this paper, the APCA of China’s eight main apple-producing provinces from 2013 to 2022 was measured by using a aggregate comparative advantage index. The spatio-temporal dynamic evolution characteristics of APCA were revealed by adopted Arc GIS and kernel density estimation method. Second, the transfer probabilities of different types of APCA were predicted by empolyed traditional and spatial Markov chains. Finally, the driving mechanism of APCA is explored with the panel quantile model.

**Results:**

1) The average value of APCA of the main producing provinces increased from 1.330 in 2013 to 1.419 in 2022. 2) The probabilities of provinces with low, primary and middle level of advantage jumping to the next level are 31.58%, 16.67% and 11.76%, respectively. When the spatial lag type is high-level advantage, the probability of stabilization of the low-level advantage decreases from 68.42% to 0.00%. 3) Nonfarm payrolls have the largest dampening effect at the 40% quantile.

**Conclusions:**

1) Temporally, APCA shows a trend of slow growth, ups and downs. Spatially, APCA shows a distribution pattern of “west high, east low”. 2) APCA mainly shifted sequentially between neighbouring ranks. Besides, the change of APCA had significant spatial spillover effect, and highly advantage provinces featured more prominent proactive spillovers. 3) There is significant heterogeneity among the influencing factors.

## Introduction

Apple is a globally vital fruit cash crop and agricultural product with high-value attributes, playing an essential role in rural poverty alleviation and the sustainability of agriculture in many developing countries [1]. For many years, China has always been ranked as the world’s top producer and consumer of apples, with apple acreage and production consistently topping the charts in the global arena. In 2022, the total global apple production was 82.9340 million tonnes. Among them, China’s annual output reached 47,5718 million tonnes, topping the world and occupying more than 50% of the global share [2]. Since China’s reform and opening-up, the rapid development of the apple industry has injected lasting vitality and momentum into the growth of its agricultural and rural economy, and has become an essential pillar industry for economic development and a model for the promotion of agricultural structural adjustment [3].

China’s apple growing areas are widely distributed. Currently, apple cultivation areas are primarily clustered in two major production regions: the Loess Plateau and Bohai Bay [4]. In 2022, the proportion of apple growing acreage in the Loess Plateau and Bohai Rim regions accounted for 52% and 33% of the total national acreage, respectively [5]. In terms of provincial distribution, it mainly includes eight main producing provinces, namely Shaanxi, Shandong, Henan, Shanxi, Hebei, Gansu, Liaoning, and Ningxia. It is estimated that, as the world’s largest concentrated and continuous apple planting area, Shaanxi Province, through the development of apple planting industry, pulled nearly 10 million of the province’s agricultural population employment, and drove 45 of the province’s national apple base counties of the farmers’ incomes are more than 10,000 yuan [6]. Therefore, the eight main producing provinces not only ensure the main source of apples in China, but also raise the farmers’ earnings and enhance their living standards [7].

Nonetheless, the speedy pace of urbanization and industrialization poses a series of challenges to apple production in the main producing provinces [3]. Specifically, based on the demand side, driven by the upgrading of consumer demand, there is a vigorous consumer demand for high-quality apples, branded apples and their processed products, which brings brand-new challenges and opportunities for value enhancement and industrial transformation to apple production in the main producing provinces [1]. Based on the supply side, the main producing provinces are facing problems such as the replacement of old orchards, the transfer of young and strong labour, the increase in the price of agricultural materials year by year, the rapid increase in the cost of orchard production, the decline in apple production efficiency [8].

Notably, a widening range of literature emphasises the crucial function of comparative advantages in crop production [9–12]. The organization of agricultural production according to comparative advantage in production can raise the allocation efficiency of resources, optimize the variety structure [13], and can promote the specialized division of labour, upgrade the production structure, and then increase the overall income for farmers [1]. Thus, research on the APCA is not only favourable for promoting clustering in apple production towards advantageous production areas, optimizing the production layout of apples, and improving the total factor productivity of apple production, but also beneficial to the main apple-producing provinces to achieve stable yield, high yield, quality and efficiency, and to offer a useful reference for the quality growth of apple sector in China.

The remainder of this study is organized as follows: The Literature Review section describes the relevant literature and the Materials and Methods section outlines the research materials and data used in this paper. The Results section presents the findings of this paper. The Discussion section contains a discussion and analysis of the content and results of this paper in relation to existing studies. The Conclusion section summarizes the main conclusions, limitations and future research directions of this paper. The Policy Implications section offers policy recommendations based on the findings of this paper.

## Literature review

### Agricultural products production comparative advantage

Agricultural products production comparative advantage refers to the difference in opportunity costs in the production and marketing of agricultural products in a country or region due to variations in resource endowments such as elements of natural conditions, labour factors, etc [14], which reflects the productivity and competitiveness of agricultural products, and offers a reference basis for the formulation of government policies. At present, there are more research methods on agricultural products production comparative advantage, mainly including comparative advantage index [15], policy analytical matrix [16], resource cost method [17] and the integrated comparative advantage index [18]. Among them, the integrated comparative advantage index captures the level of development of production scale and efficiency advantages and performs most prominently [12]. The research field of agricultural products production comparative advantage covers a wide range of areas [18]. But unfortunately, most of the existing literature focuses on annual crops such as grain, and only a few scholars have studied the production comparative advantage of perennial crops such as apples. For example, Zhang, Chang [19] combined a variety of methods, such as apple concentration index and comparative advantage index, to reveal five stages of apple production in China. Song, Zhang [7] validated the spatial effect of APCA by utilizing the spatial Durbin model. Hence, there are not many systematic studies on the APCA in current academia.

### Apple production

Based on enhancing the international competitiveness of apple production, Muder, Garming [20] assessed the apple value chain of nine key apple-producing nations across Europe and found that climate risk increases the cost of disaster mitigation measures in apple production. Eyduran, Akın [21] with the help of an ARIMA model forecasted 2020–2025 growth trend of Turkey’s apple production. With the tightening of resource and environmental constraints on apple cultivation, some scholars have launched an exploration of total factor productivity in apples [22], and gradually extended in depth to apple production energy use rate [23], technical efficiency of apple production [24] and so on. Meanwhile, more and more studies have examined the impact of farmers’ behaviour on apple production from a micro perspective, such as production outsourcing [19], green production willingness [25], good agricultural practices [23] and other factors.

### Apple production comparative advantage

In the existing research on the APCA, previous scholars mainly applied the comprehensive comparative advantage index to explore the characteristics of the production layout of apples, and it has been shown that the production layout of Chinese apples has been gradually clustered in the two advantageous areas of the Bohai Bay Rim and the Loess Plateau [19,26], and has shown the development trend of “westward shift and northward expansion” [26]. In addition, using the improved comprehensive comparative advantage index method, Li [27] analyzed the trend of comparative advantage changes in the main apple producing areas. Utilizing the comprehensive comparative advantage index, Zhang [28] evaluated the layout of APCA in China, whose study found a lack of consistency between efficiency and scale comparative advantage in some provinces and regions. On the basis of analyzing the center of gravity of main production areas, Wang [3] further employed the APCA to fully analyze the spatial layout of apple production in China. In terms of the driving factors of APCA, Zhang, Chang [19] explored the effects of temperature, rainfall, and transportation infrastructure on APCA. By means of spatial panel modeling, Song et al [7] verified the significant effects of the number of laborers and planting costs on APCA.

In summary, although existing studies have achieved some valuable research results in the APCA and the layout of production areas, there are still research gaps that need to be filled: first, existing empirical studies on the APCA have neglected the influence of spatial factors, resulting in biased results, and meanwhile, most of them have focused on describing the characteristics of the spatial-temporal evolution, and lacked analysis of the long-term development trend incorporating spatial factors. Second, the available studies have ignored the influence of different factors on the APCA under the perspective of heterogeneity, thus the limitation of mean regression exists.

In order to make up for the shortcomings of the above studies, this paper updated the time of APCA data sources, measured the production comparative advantage of China’s eight major apple-producing provinces from 2013 to 2022 by utilizing the comprehensive comparative advantage index, applied Arc GIS and kernel density estimation approaches to reveal the dynamics of the spatio-temporal distribution of APCA, predicted the trend of APCA by means of traditional and spatial Markov chains, and examined the extent to which different influencing factors under the perspective of heterogeneity by adopting the panel quantile regression method, so as to offer a theoretical basis to formulate a scientific and effective policies on apple production and to realize the high-quality development of apple sector.

The possible marginal contributions from this research are listed below: First, in the measurement of the spatio-temporal dynamic evolution characteristics, this study not only updated the data source, but also applied a new measurement method, i.e., three-dimensional kernel density estimation, to efficiently reveal the dynamic evolution law of APCA in the main producing provinces. Secondly, unlike previous research, this paper forecasted the development trend of APCA. In this process, this paper considered the influence of spatial factors and further constructed the spatial Markov transfer probability matrix to verify the influence of spatial relationships on the development trend of APCA. Finally, this study introduced the panel quantile model into the study of production comparative advantage influencing factors for the first time. The effect of different socio-economic factors on APCA in the major producing provinces was investigated, controlling for natural conditions. In particular, the panel quantile model examined the differences in the influencing factors at different levels of APCA with heterogeneity characteristics, which not only could better control individual heterogeneity, but also could analyse the marginal effect of each influencing factor on APCA at a specific quantile, which is, to a certain extent, a useful supplement to the existing research methodology on the influencing factors of APCA.

## Materials and methods

### Research materials

#### Study area

**Fig 1:**
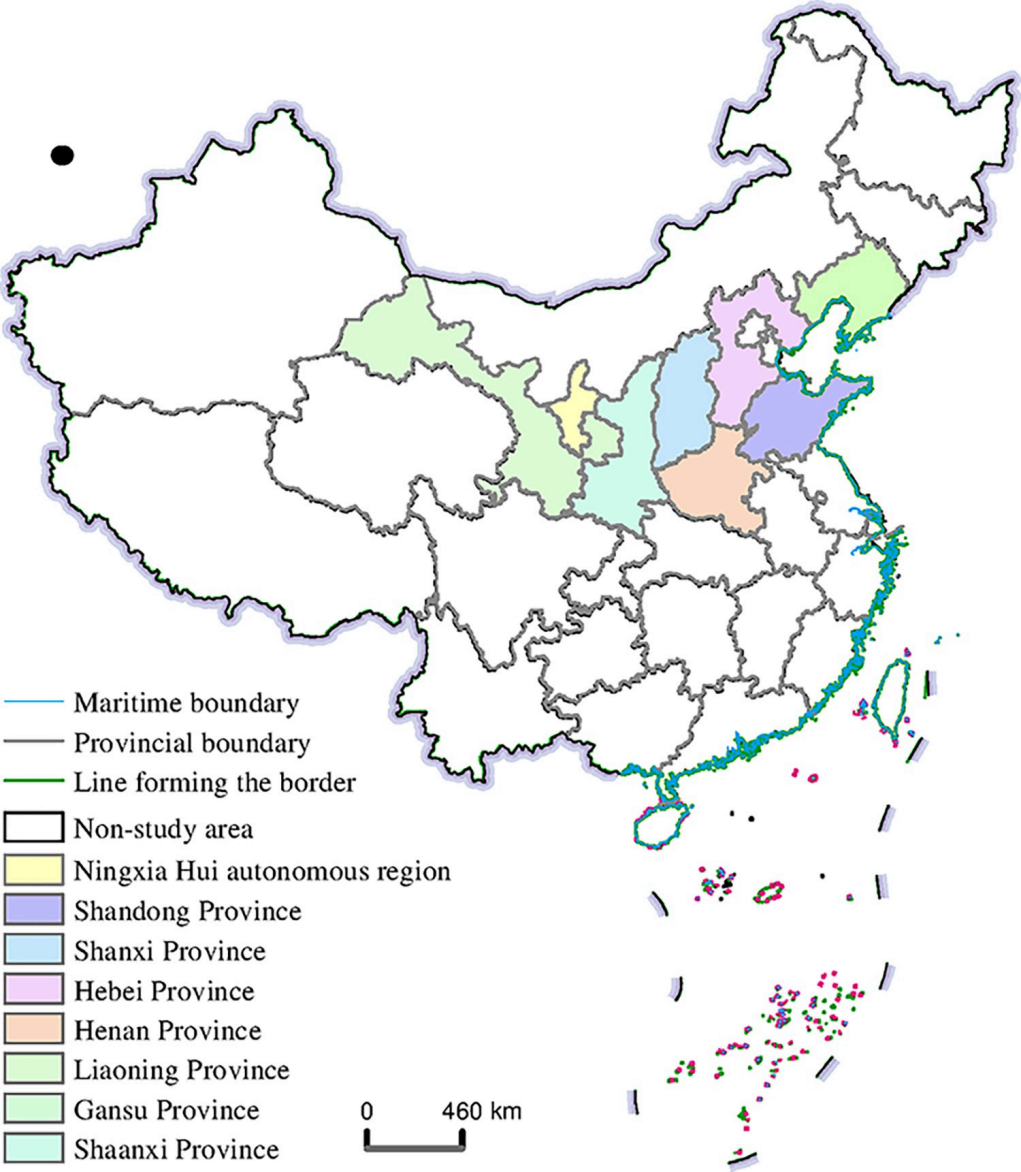
Study area (Map from the Ministry of Natural Resources of China, cartographic license: GS (2020) 4619).

China’s apple cultivation area is vast, with a large latitude and longitude span. Among them, the main apple producing areas are mainly concentrated in the Bohai Bay Rim producing areas, mainly in Shandong, Hebei and Liaoning, and the Loess Plateau producing areas, mainly in Henan, Gansu, Shaanxi, Shanxi and Ningxia provinces. Over the years, the two major apple-producing regions have accounted for more than 80% of China’s total apple production, supporting China’s dominant position in the global production of apples, which is typical and representative. Thus, this paper selected the eight main producing provinces of China’s major apple-producing regions from 2013 to 2022 as research object (Fig 1).

#### Influencing factors of APCA

In this paper, the factors of average annual temperature, precipitation and sunshine hours, which reflect natural conditions, are used as control variables [3]. On the basis of controlling the influence of natural factors, taking into account the characteristics of apple production and data availability, this paper selects influencing factors from five aspects, namely, agricultural infrastructure, agricultural science and technology level, governmental agricultural intervention, economic efficiency factors, and labour force factors, respectively, to analyse the extent to which different socio-economic drivers affect the comparative advantage in apple production. The details are provided in Table 1.

**Table 1:** Influencing factors of APCA.

Dimension	Variable	Measurement methods	Symbol
Agricultural Infrastructure	Effective irrigation area for apples	Effective irrigated area for apples = (area planted with apples/total area planted with crops) × effective irrigated area for crops	Ln_AI
Agricultural Science and Technology Level	Agricultural science and technology patents	Number of three types of patents in agriculture in China National Knowledge Infrastructure patent database	Ln_AT
Government Agricultural Expenditure	Expenditure on agriculture, forestry and water affairs	Collected from China Rural Statistics Yearbook	Ln_GOV
Economic efficiency	Output value of apple production	Collected from China Rural Statistics Yearbook	Ln_EB
Labor Force	Rural non-farm employment	Collected from China Rural Statistics Yearbook	Ln_Rl

#### Data sources

Considering that the data involved in this paper have not yet been released in 2023 and 2024 as well as the availability and applicability of the data, this paper chooses 2013–2022 as the study period. The data of apple planted area, fruit planted area, apple yield, fruit yield, and effective irrigated area of crops involved in this paper are mainly collected from the China Statistical Yearbook and China Rural Statistical Yearbook of the current year. The data on annual average temperature, precipitation and sunshine hours are mainly collected from provincial statistical yearbooks. Data on agricultural, forestry and water expenditures, apple production output value, and rural non-farm employment were compiled from the China Rural Statistical Yearbook. The data on agricultural science and technology patents come from the China National Knowledge Infrastructure Patent Database.

In order to avoid the influence of abnormal samples and ensure the stability of the samples, this paper processed the raw data according to the following two steps: 1) The data with a small number of missing numbers were supplemented by the Statistical Bulletin of Provinces and the interpolation method. 2) In order to eliminate the interference of outliers on the regression results and ensure the accuracy of the data, this paper took logarithms of the main variables that need to be regression analyzed.

After the above processing, this paper finally forms the panel data of 8 China’s main apple-producing provinces from 2013 to 2022 as the overall research sample.

### Research methodology

#### Comprehensive comparative advantage index

This paper picked the scientifically sound and representative Aggregate Comparative Advantage Index (AAI) to calculate APCA, which usually consists of efficiency comparative advantage index (EAI) and scale comparative advantage index (SAI) [10]. The AAI is a comprehensive measure of the relative comparative advantage of a region in the production of a particular agricultural product, based on both the relative productivity advantage and the scale advantage determined by a combination of factors such as market, technology, cropping system, climate and geographic location. The larger the AAI value, the more obvious the advantage. The SAI reflects crops’ comparative advantage from scale of production and degree of specialization, embodying the outcome of the combination of market demand, cropping systems, policy support, labour and material inputs and resource endowments on production. The EAI captures crops’ comparative advantages in terms of resource-contained productivity, which is a comprehensive reflection of agricultural resource conditions, scientific and technological level, material input level and other factors. The specific formula is as follows:

$$SAI_{it}=GS_{it}/GS_{i}÷GS_{t}/GS$$ (1)

$$EAI_{it}=VS_{it}/VS_{i}÷VS_{t}/VS$$ (2)

$$AAI_{it}=\sqrt{SAI_{\mathrm{it}}\cdot EAI_{\mathrm{it}}}$$ (3)

Among them, the scale advantage index *SAI*_*it*_, expressed as the ratio of *GS*_it_/*Gs*_i_ (area of apples in place i/area of fruits in place i) to *GS*_t_/GS (national apples/national fruits area); and the efficiency advantage index *EAI*_it_, expressed as the ratio of *VS*_it_/*VS* (yields of apples in place i/yields of fruits in place i) to *VS*_t_/*VS* (national apples yields/national fruits yields ratio); *AAI*_it_ is the geometric mean of EAIit *EAI*_it_ and *SAI*_it_, with *AAI*_it_>1 indicating the integrated comparative advantage of apple yield in place i exceeds the national average, and vice versa.

#### Kernel density estimation

Kernel density estimation fits the function’s distribution from the characteristics of the data itself, avoiding errors that may be caused by artificially setting the form of the function [29]. In this study, kernel density estimation was employed to characterize dynamic evolution in APCA from multiple perspectives, such as the location of the curve map distribution of APCA, the shape of the curve and the change in peak value. The detailed formula is given below:

$$f(x)=\frac{1}{nh}\sum_{i=1}^{n}k(\frac{X_{i}-x}{h})$$ (4)

Where: *X*_*i*_ is the ith independent and identically distributed observation; *x* is the mean of the observations; $k(\frac{X_{i}-x}{h})$ is the Gaussian kernel function; *n* is the amount of sample observations; and *h* is the bandwidth.

#### Traditional Markov chains

A traditional Markov chain represent discrete Markov processes in respect to time and state, reflects the APCA state for which each individual within the area and its the likelihood of shifting up or down [30]. Firstly, this paper discretised the APCA data into k types, subsequently calculated the probability distribution and transfer changes for each state type, and finally derived the developmental evolutionary law of APCA. The specific calculation steps are as follows:

$$M=(\begin{array}{cccc} m_{11} & m_{12} & \ldots & m_{1j}\\ m_{21} & m_{22} & \ldots & m_{2j}\\ \ldots & \ldots & \ldots & \ldots \\ m_{i1} & m_{i2} & \ldots & m_{ij} \end{array})$$ (5)

Where: *M* denotes the Markov transfer probability matrix for *k*×*k*; and the *m*_*ij*_ element denotes the probability that a province of type *i* in year *t* transfers to type *j* in year *t*+1 and is estimated using *m*_*ij*_ = *n*_*ij*_/*n*_*i*_.

#### Spatial Markov chain

Spatial Markov chain is an innovative model that adds "spatial lag" to traditional Markov chain, which is able to deeply analyse the evolution pattern of panel data in time evolution and spatial context [30]. In this paper, conditional on the type of spatial lag in the first year, the traditional transfer probability matrix M of traditional k×k order state was resolved into k kinds of k×k transfer conditional probability matrices for the purpose of analyzing probabilistic of APCA occurring in various neighborhood contexts. The specific calculation steps are as follows:

$$Lag_{a}=\sum_{b=1}^{n}\left(Y_{b}W_{ab}\right)$$ (6)

where: *Lag*_*a*_ is the spatial lag value of province *a*; *Y*_*b*_ is the observed value of province *b*; and *W*_*ab*_ is the spatial weight matrix (neighbouring is 1 and vice versa is 0).

#### Panel quantile regression

Most of the studies using ordinary panel data models for heterogeneity analysis are conducted based on the assumption of mean regression, and the regression results can only portray the structure of heterogeneity at the mean level. While heterogeneity analysis approaches based on panel quantile model can reflect more comprehensively the regression relationship between variables at different quartiles from the perspective of the conditional distribution of the explanatory variables and identify their heterogeneity structure [31]. Therefore, employing panel quantile regression, this paper analyzed how the factors of various quantile points affect APCA under the heterogeneity perspective, avoiding idealistic model of mean regression, providing more information between observed variables and making the research conclusions more reliable. The concrete calculation steps are listed below:

$$Q_{Y_{it}}(\tau |X_{it})=a_{i}+X_{it}^{T}\beta (\tau ),(i=1,2,\cdots ,n;t=1,2,\cdots ,T)$$ (7)

Where: $Q_{Y_{it}}$ is the conditional quantile function of APCA; *Y*_*it*_ is APCA; *a*_*i*_ is the constant term; *X*_*it*_ is the matrix of explanatory variables; *β*(*τ*) is the impact coefficient under the *T* quantile; and *T* is the quantile point set in this paper.


$$\beta (\theta )=\underset{\left(\alpha ,\beta \right)}{\min}\sum_{k-1}^{q}\sum_{i=1}^{n}\sum_{t=1}^{T}w_{k}\rho_{\tau_{k}}[Y_{it}-a_{i}-X_{it}^{T}\beta (\tau_{k})]$$ (8)

Where: *β*(*θ*) is the influence coefficient; *q* is the number of quantile arrays; *k* is the *k* th group of quantile arrays; $\rho_{\tau_{k}}$ is the quantile loss function; *w*_*k*_ is the weight coefficient of the *k* th quantile; *β*(*τ*_*k*_) is the influence coefficient of the *k* th quantile.

## Results

The flow of data analysis in the results section of this paper is as follows: first, based on measuring the APCA of the eight main apple-producing provinces in China from 2013 to 2022 by using the AAI index, this paper demonstrated the changes in the mean values of APCA, SAI, and EAI of the eight main apple-producing provinces with the help of a line graph. Secondly, this paper revealed the dynamic evolution law of APCA in the main producing provinces with the help of kernel density estimation method. Third, this paper applied Arc GIS to present the spatio-temporal pattern characteristics of APCA, SAI, and EAI. Fourth, this paper examined the development trend of APCA in China’s main apple-producing provinces by utilizing traditional Markov chains, and verified the influence of spatial relationships on the development trend of APCA by constructing a spatial Markov transfer probability matrix. Finally, this paper adopted the panel quantile regression model to examine the degree of influence of different influencing factors on APCA under the perspective of heterogeneity.

### Temporal-spatial dynamic evolution of APCA

#### Characteristics of temporal evolution of APCA

**Fig 2:**
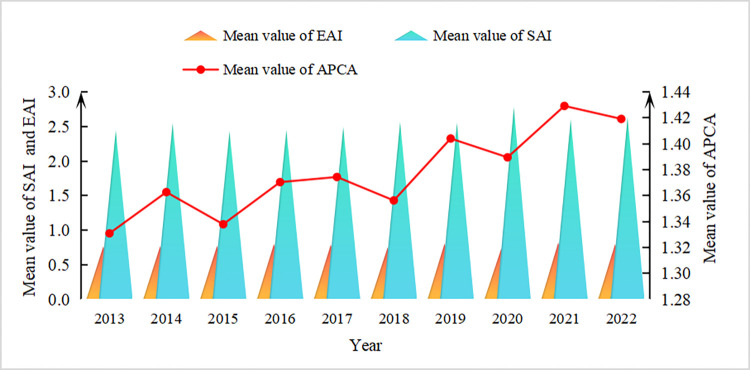
Temporal trends in APCA.

It is evident from Fig 2 that the fluctuation of APCA in the major producing provinces over our study period is significant, rising to 1.419 by 2022 from 1.330 by 2013, showing a trend of slow rise, high and low ups and downs. Specifically, the SAI in apple production was significantly better than EAI over the long term, which indicated that there was a bottleneck in the main producing provinces to a certain extent that the planting scale is large but the yield per unit were lagging behind the expansion of the planting scale.

**Fig 3:**
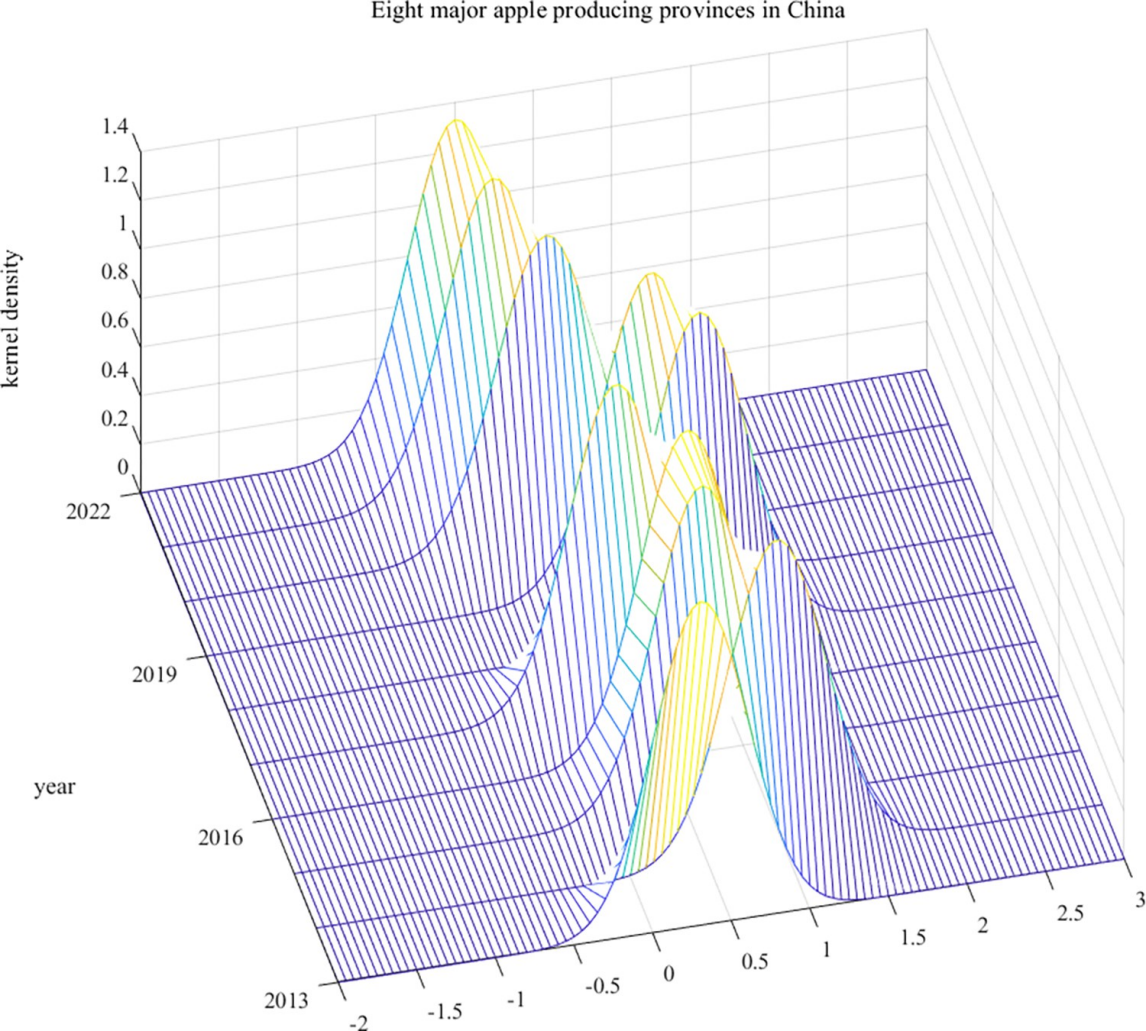
Three-dimensional kernel density of APCA.

To demonstrate the dynamical evolutionary trajectory for APCA in the main producing provinces, this paper further carried out kernel density analysis (Fig 3). 1) Distribution location: Taking 2019 as the boundary, the kernel density curve showed an obvious rightward shifting trend from 2013–2019, during which APCA in the main producing provinces had been effectively improved. In 2019, the kernel density curve presented a clear leftward shift, signalling a decline in APCA values in the main producing provinces. This mirrors the adverse effect of COVID-19 upon fruit industries such as apples. Under the influence of the epidemic, it is difficult to get efficient and comprehensive protection for labour and agricultural materials needed for apple production in the main producing provinces, which cast a shadow over the improvement of APCA in the main producing provinces. 2) Curve shape: the right tail of kernel density curve spread rightwards, indicating that there were differences in APCA among the main producing provinces, and there were individual "very high, very low" provinces. 3) Change in peak value: The breadth of kernel density curve was gradually widening and the peak value is rising, meaning that the difference in APCA between the main producing provinces tends to widen.

#### Spatial evolution characteristics of APCA

**Fig 4:**
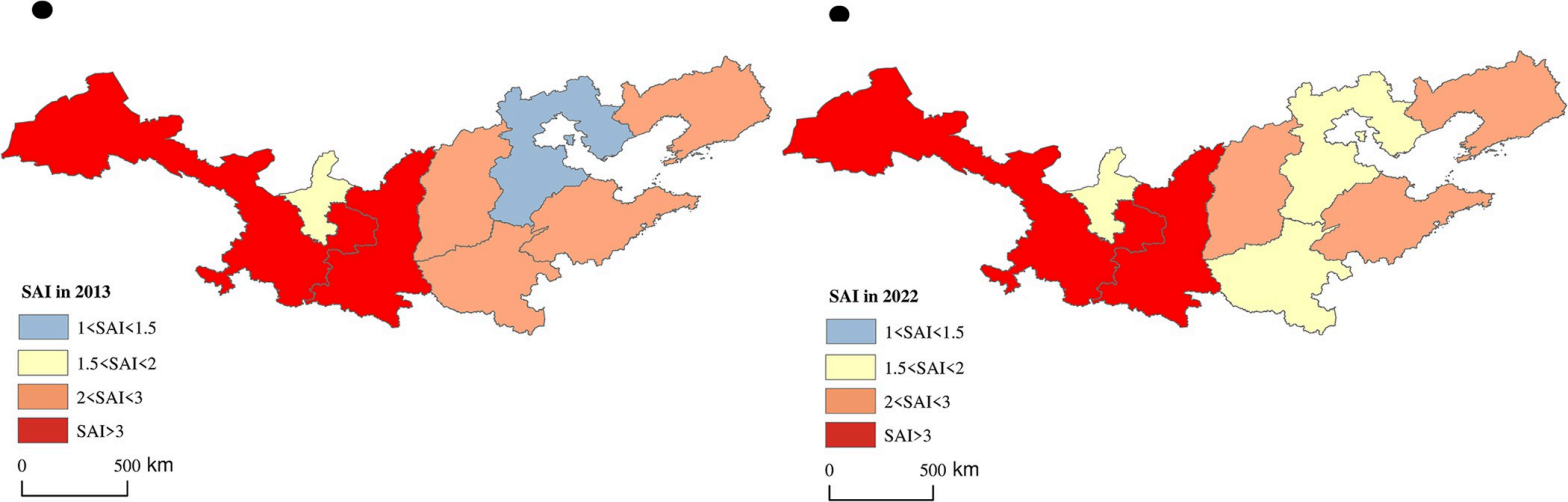
Spatial distribution of SAI (Map from the Ministry of Natural Resources of China, cartographic license: GS (2020) 4619).

**Fig 5:**
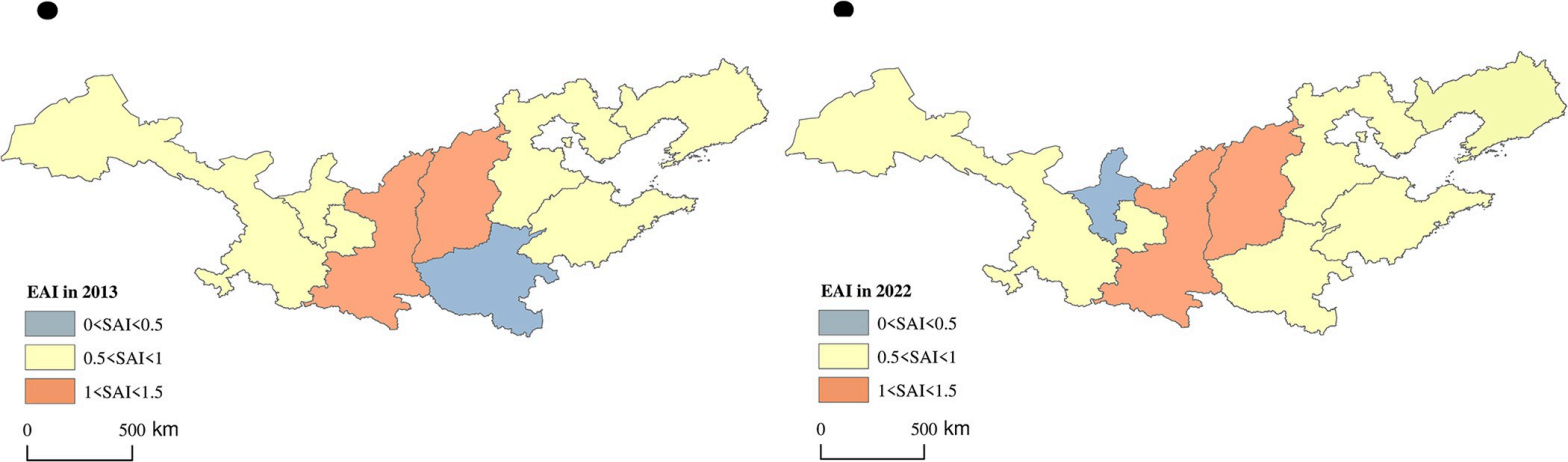
Spatial distribution of EAI (Map from the Ministry of Natural Resources of China, cartographic license: GS (2020) 4619).

By means of Arc GIS, the spatial visualization analysis results of APCA, SAI and EAI of apple main producing provinces in 2013 and 2022 were obtained. SAI exhibited a "west-high east-low" spatial pattern (Fig 4). Among the main-producing provinces, Shaanxi and Gansu had always been in the leading position, with absolute advantages in apple cultivation area, while Ningxia had always been in the last position. The EAI showed a "middle high, surrounded by low" spatial distribution (Fig 5). In 2013 and 2022, only Shaanxi and Shanxi provinces had EAI greater than 1. The rest of the production areas had EAI less than 1. The reason for this may be that the improvement of efficiency mainly depends on factors such as scientific and technological development, variety optimization and management level, etc. In other words, the improvement of EAI is a long-term "quality" issue rather than a short-term "quantity" issue.

**Fig 6:**
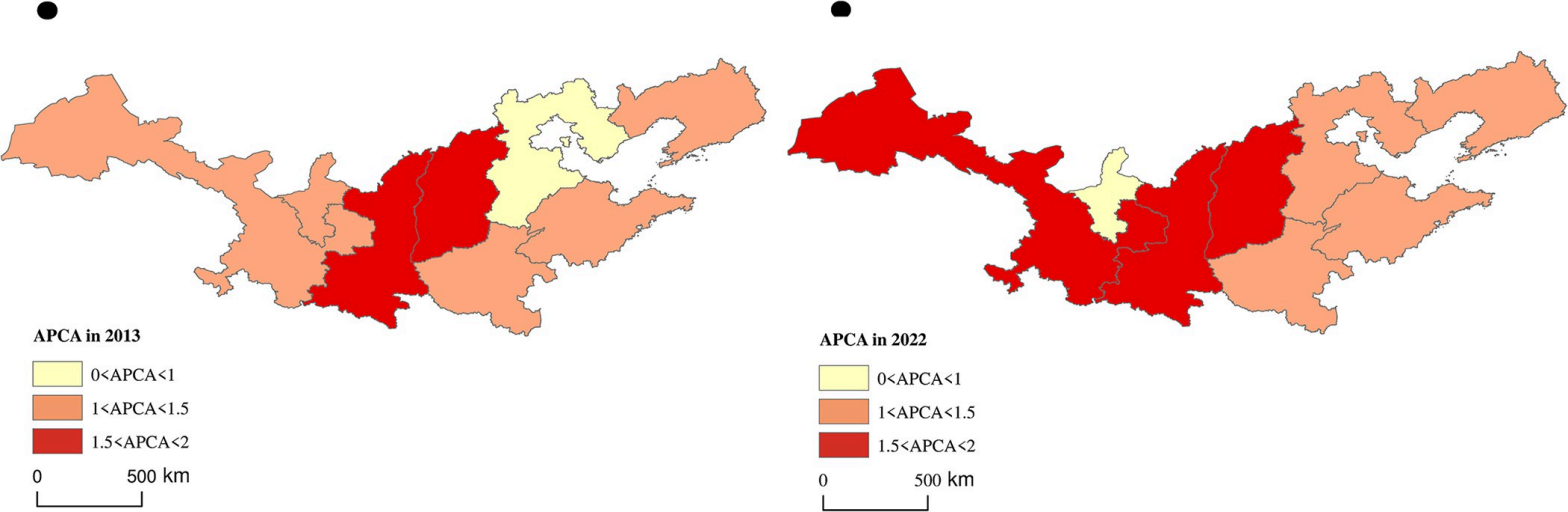
Spatial distribution of APCA (Map from the Ministry of Natural Resources of China, cartographic license: GS (2020) 4619).

The APCA displayed distribution pattern of "Loess Plateau producing areas leading and Bohai Bay producing areas relatively lagging behind" (Fig 6), reflecting the obvious spatial differences in APCA. Among them, Gansu Province stood out. However, it is worth noting that Ningxia had gradually lost its original comparative advantage compared with other producing areas within the Loess Plateau. Hebei Province, on the other hand, had gradually risen to become a production area with higher production comparative advantage, narrowing the differences in comparative advantage between the Bohai Bay producing areas.

### Trend prediction of APCA

#### Traditional Markov chain

This paper used Markov chain transfer matrix to forecast the trend of change in APCA in China’s main apple-producing provinces. The Markov transfer matrix describe the probability of a system transferring from one level state to another. Therefore, we need to divide the APCA using the quartile method to observe the probability of transferring the APCA from different levels of development to another. Quartiles are commonly used. Quartile method is a type of quartile in statistics where all the values are arranged from the smallest to the largest and divided into four equal parts, and the values at the position of the three dividing points are the quartiles. Therefore, in this paper, the quartile method was used to arrange the APCA of the eight major producing provinces from smallest to largest and divide them into four equal parts, and defined them from low to high as low-level advantage (LA), primary advantage (PA), medium-level advantage (MA), and high-level advantage, respectively, which represented four different levels of development grades of the APCA from low to high. On this basis, the transfer probability matrix of APCA in major apple-producing provinces in China was obtained based on the states of the four APCA classes with a one-year lag.

By comparing the transfer probabilities on the diagonal with those on the off-diagonal in Table 2, we can find that the transfer probabilities on the main diagonal LA→LA (68.42%), PA→PA (61.11%), MA→MA (82.35%) and HA→HA (88.89%) are significantly higher than the off-diagonal transfer probabilities. It shows that the probability of the four levels of APCA remaining unchanged is higher in the major apple-producing provinces of China. Secondly, rank shifts in APCA occur mainly between neighbouring advantage types, which demonstrates that the enhancement of APCA is not achieved overnight, but is a gradual development process. Specifically, the probabilities of provinces at the lower, primary and intermediate levels of advantage leaping to the next level are 31.58%, 16.67% and 11.76%, respectively, which implies that there is room for the provinces’ APCA to rise further, with a trend of leapfrogging to a higher level of advantage. The probabilities of provinces with primary, intermediate and advanced advantages declining to the next level are 22.22%, 5.88% and 11.11% respectively. Relatively speaking, the probability of shifting upwards in the APCA is higher in the major apple-producing provinces, while the probability of shifting downwards is relatively smaller. Therefore, the main apple-producing provinces should be prudent in avoiding the risks caused by the damage to the APCA, and actively stabilize the APCA and endeavour to jump upward to the upper level of comparative advantage.

**Table 2:** Traditional Markov transfer probability matrices.

	LA	PA	MA	HA	n
LA	0.6842	0.3158	0.0000	0.0000	19
PA	0.2222	0.6111	0.1667	0.0000	18
MA	0.0000	0.0588	0.8235	0.1176	17
HA	0.0000	0.0000	0.1111	0.8889	18

#### Spatial Markov chain

Our paper further constructed a spatial Markov transfer probability matrix to verify the influence of spatial relationships on the development trend of APCA. It can be seen from Table 3 that there are differences in the four transfer probability matrices for the four spatial lag conditions, indicating the probability of APCA switching varies from neighbouring region, and that there is some spatial geographic dependence. Secondly, the presence of non-zero values on either side from the diagonal of the spatial Markov transfer matrix at different spatial lags demonstrates that inter-regional APCA hierarchical shifts are likely to occur mainly between neighbouring types. Type evolution has instability. Thirdly, affected by the spatial lag state, the Markov transfer probability of APCA changes significantly, and the neighbouring region has a notable spatial spillover effect on local region. When the type of spatial lag is high-level advantage, the stability probability of low-level advantage decreases from 68.42% to 0.00%, showing that the stable state of APCA displays an obvious wavering trend, and when neighbouring high-level advantage regions, the region with low APCA is likely to have a breakthrough and jump to the next level. Furthermore, it is noteworthy the steady-state probability of high-level advantage is 0.00% when the low-level advantage is a neighbouring region, suggesting that it is also highly susceptible to the negative spillover effect of the neighbouring region of low-level advantage.

**Table 3:** Spatial Markov transfer probability matrix.

Type of neighbouring region		LA	PA	MA	HA	n
LA	LA	0.0000	0.0000	0.0000	0.0000	0
	PA	0.0000	0.7000	0.3000	0.0000	10
	MA	0.0000	0.2000	0.8000	0.0000	5
	HA	0.0000	0.0000	0.0000	0.0000	0
PA	LA	1.0000	0.0000	0.0000	0.0000	4
	PA	0.0000	0.0000	0.0000	0.0000	0
	MA	0.0000	0.0000	0.8571	0.1429	7
	HA	0.0000	0.0000	0.1429	0.8750	14
MA	LA	0.6923	0.3077	0.0000	0.0000	13
	PA	0.2000	0.8000	0.0000	0.0000	5
	MA	0.0000	0.0000	0.8000	0.2000	5
	HA	0.0000	0.0000	0.0000	1.0000	4
HA	LA	0.0000	1.0000	0.0000	0.0000	2
	PA	1.0000	0.4000	0.0000	0.0000	3
	MA	0.0000	0.0000	0.0000	0.0000	0
	HA	0.0000	0.0000	0.0000	0.0000	0

#### Influencing factors of APCA

To select a specific form of the model, we screened tests for Pooled OLS model (POLS), Random Effects model (RE) and Fixed Effects model (FE). Firstly, the FE and POLS were screened utilizing the F-statistic test. The P-value result was 0.000, so the original hypothesis was strongly rejected, and there was a clear advantage of using FE over POLS. Secondly, the Hausman test was applied to compare the random and fixed effects and the p-value was 0.000, so the original hypothesis was rejected and the FE should be used. In short, this paper chose a fixed-effects quantile regression model, which evaluated by using the Markov Chain Monte Carlo (MCMC) method. In the panel quantile model, this paper chose conventional 20%, 40%, 60% and 80% quantile points for estimation, which is beneficial for comparing the differences of the influencing factors under different APCA. Moreover, in order to make comparisons with the average regression coefficients of conventional panel data models, this paper comparatively estimated the FE. The empirical analyses in this paper were all completed by stata16, and the outcomes are reported in Table 4.

**Table 4:** Fixed effects and panel quantile regression results.

Variables	Fixed Effects APCA	QR_20 APCA	QR_40 APCA	QR_60 APCA	QR_80 APCA
Ln_GOV	0.0708 (0.04800)	-0.2590*** (0.00003)	-0.1950*** (0.00013)	-0.1490*** (0.00021)	-0.0843*** (0.00044)
Ln_EB	0.1130*** (0.03090)	0.1540*** (0.00001)	0.0936*** (0.00005)	0.0607*** (0.00012)	0.0467*** (0.00010)
Ln_AT	-0.0574** (0.02560)	0.0596*** (0.00001)	0.0510*** (0.00006)	0.0274*** (0.00008)	0.0208*** (0.00024)
Ln_AI	0.2210** (0.08970)	0.3090*** (0.00001)	0.3740*** (0.00008)	0.3950*** (0.00018)	0.3940*** (0.00026)
Ln_Rl	-0.1300*** (0.03210)	-0.1800*** (0.00001)	-0.1900*** (0.00003)	-0.1880*** (0.00005)	-0.1810*** (0.00022)
Control variable	Yes	Yes	Yes	Yes	Yes
Year FE	Yes	Yes	Yes	Yes	Yes
Province FE	Yes	Yes	Yes	Yes	Yes
N	80	80	80	80	80

Notes:Standard errors in parentheses.

*** p<0.01

** p<0.05

* p<0.1.

From the regression results of FE, apple economic efficiency and effective irrigated area significantly increased APCA, indicating that economic efficiency and agricultural infrastructure were critical factors influencing the growth of APCA in major producing provinces and affecting the modernisation of the apple sector. Agricultural technological advancement and non-farm employment were the main factors inhibiting the increase in APCA, which may be due to the following reasons, respectively: Firstly, compared with apples, food crops are easier to be operated on a large scale. Agricultural technological advancement is more likely to substitute production factors for food crops. Secondly, apple is a labour-intensive crop, and along with the rapid development of urbanization in China, non-farm employment opportunities in neighbouring towns and cities have increased, which has in turn gradually increased the opportunity cost for farmers to engage in apple production. In this context, the continuous transfer of fruit farmers’ labour to industries in non-farming areas, especially the large number of young labourers moving to the urban non-farming sector, is a serious constraint to the sustainable development of the APCA.

From the trend of the coefficient change at the interquartile level, the estimated coefficient of government agricultural expenditure gradually decreased from -0.2590 to -0.0843 with the change of the interquartile point, meaning that the negative effect of government agricultural expenditure on the main producing provinces with higher level of APCA was smaller than that of the provinces with lower APCA. The reason for this is that there is a certain substitution relationship between food crop cultivation and apple cultivation, while the positive effect of government agricultural expenditure on stabilizing traditional food crop cultivation is more far-reaching and extensive. Therefore, for regions with low APCA with poor conditions for apple cultivation, the substitution effect of food crops for apples will increase as government spending on agriculture increases, which will hinder the comparative advantage of apple production more significantly.

The estimated coefficients of apple economic efficiency and agricultural science and technology advancement on APCA gradually decreased from 0.1540 and 0.0596 to 0.0467 and 0.0208, respectively, indicating that economic efficiency and agricultural science and technology had a greater impact on provinces with low APCA than on provinces with high APCA. The probable causes could be the following: for one, the higher economic benefits of apples for regions with low APCA, the higher comparative returns to apples, and the more attractive apple cultivation is to other farmers. Secondly, compared with high-advantage areas, low advantage provinces lack experience in apple cultivation and have weak resource endowments. Thus, the improvement of agricultural science and technology can reduce the risk of apple production to a greater extent, thus increasing the planting enthusiasm of fruit farmers.

The estimated coefficients of effective irrigated area of apple at each quantile point were larger than the mean regression coefficient of FE, and their coefficients were significantly different. The estimated coefficients gradually increased from 0.3090 to 0.3940 with the change of quartiles, indicating that the promotion effect of irrigation conditions on APCA was more obvious in provinces with higher APCA.

The estimated coefficients of non-farm employment were significantly positive at all quartiles and were larger than the FE regression coefficients, signifying that the ordinary FE regression underestimated the inhibitory effect of non-farm employment on APCA. Therefore, it is imperative to introduce relevant preferential policies to attract farmers to contract orchard production and operation in the main apple-producing provinces where labour mobility is high and aging is serious.

## Discussion

### Discussion of spatio-temporal dynamic evolution

In terms of the spatio-temporal dynamic analysis of APCA, unlike the traditional research methods, this paper applies a new measurement method, namely, three-dimensional kernel density estimation, which effectively reveals the dynamic evolution law of APCA. In addition, unlike the conclusions of existing studies, the kernel density curves of APCA in the main producing provinces gradually become wider in width and increase in peak value, which indicates that the gap of APCA between the main producing provinces tends to widen, which is not conducive to the formation of the characteristic apple production areas. Meanwhile, this paper finds that only Shaanxi and Shanxi provinces have EAI greater than 1. Overall, the development of SAI in the main producing provinces is still ahead of EAI, suggesting the continuity of the advantages of planting scale in the main producing provinces, and also implying that there is a long-term shackle on the improvement of yields. Elsewhere, this study found that the annual average growth rate of main production APCA was 0.716%, which maintained the growth trend. Other related studies also support this conclusion [1], which shows the validity of the findings of this paper. Moreover, the APCA of the main producing provinces in this paper presents a spatial pattern of “high in the west and low in the east”, which side by side verifies the “westward trend” of China’s apple production layout [3].

### Discussion of trend prediction results

Different from previous studies, this paper analyzes and predicts the long-term development trend of APCA on the basis of analyzing the spatio-temporal development law of APCA. In this process, this paper innovatively introduces the Markov prediction model into the analysis of APCA and finds that the probabilities of provinces with APCA in LA, PA and MA jumping up to the next level are 31.58%, 16.67% and 11.76% respectively, which implies that there is room for further rise in the APCA of each province, with the development trend of jumping up to a higher level of superiority. And considering the influence of spatial factors and further constructing the spatial Markov transfer probability matrix, this paper verifies the influence of spatial factors on the development trend of APCA. In particular, this study finds that, influenced by the spatial lag state, when the spatial lag type is HA, the LA stabilization probability decreases from 68.42% to 0.00%, indicating that provinces with lower APCA have the potential to make a breakthrough and leap to the next level when they are adjacent to the advanced advantage area.

### Discussion of influencing factors

The difference with other related literatures is that this paper adopts panel quantile regression for the first time to analyze the impact of influencing factors at different quantile points on APCA from the perspective of heterogeneity, which avoids the limitations of mean regression and provides more observational information on different time periods of the observed variables, and which extends the research methodology of influencing factors on APCA to a certain extent.

## Conclusions

The rapid development of urbanization, industrialization, globalization and complex climate change have brought a series of challenges to the apple industry in China’s main apple-producing provinces, so it is crucial to explore the evolutionary trends and influencing factors of APCA to promote the high-quality development of China’s apple industry. In this paper, we measure the APCA of China’s eight main apple-producing provinces from 2013 to 2022 using the AAI index, apply Arc GIS and kernel density estimation methods to explore the spatio-temporal dynamic evolution characteristics of the APCA, analyze the development trend of the APCA with the help of traditional and spatial Markov chains, and we examine the influence degree of different influencing factors on APCA under the heterogeneity perspective by using panel quantile regression.

Regarding the spatio-temporal dynamics of APCA, our study finds that although APCA in the main producing provinces shows a growing trend, their EAI lags behind SAI, which implies that there is a bottleneck in the main producing provinces that has a large planting scale but the yields are lagging behind the expansion of the planting scale to a certain extent. Moreover, APCA shows the distribution pattern of “Loess Plateau producing area leading and Bohai Bay producing area lagging behind”, with obvious spatial differences.

Regarding the prediction of the development trend of APCA, we find that APCA mainly tends to transfer incrementally between neighboring levels with relative stability. Considering the influence of spatial factors, this paper incorporates spatial factors into the Markov prediction model and discovers that provinces with higher levels of APCA have significant spatial spillover effects on neighboring provinces.

Regarding the influencing factors of APCA, non-farm employment is the main impediment to inhibit APCA, with the greatest inhibiting effect at the 40% quantile. This finding illustrates the importance and urgency of fostering new agricultural management subjects and establishing a professional farmer system for the sustainable development of the apple industry. Effective irrigated area of apple is the main driving factor to improve APCA. The promotion effect of irrigation conditions on APCA is more obvious in provinces with higher APCA.

Our findings are of great practical significance for promoting the modernization of agriculture in major apple-producing provinces, improving the production competitiveness of major apple-producing provinces, and promoting the sustainable development of China’s apple industry. In addition, this study has some limitations: first, considering the availability of data, this paper takes provincial-level regions as the study scale. In the future, more microscopic counties and urban areas could be selected to more accurately reflect the APCA of major producing provinces. Second, the development of production comparative advantage may be affected by multiple dimensions, and future research should comprehensively explore a wide range of influencing factors.

## Policy implications

The space for relying on expanding planting area to increase APCA is more limited, and capacity enhancement is the main way to production comparative advantage. Thus, it is necessary to improve yield per unit area to promote EAI. In the main producing provinces to promote the "leading varieties, the main technology, the main model" work to implement. Organize technical training and guidance services. Support the cultivation of a number of large-scale planting main body and socialized service main body, from point to surface to promote the large area of apple yield enhancement.

APCA has significant spatial heterogeneity and spatial dependence. Therefore, it is essential to be adept at enhancing the production comparative advantage according to the geographical pattern and resource endowment of each major producing province. Besides, smooth the flow channels of production factors among provinces, adequately exerting the radiation-driven role of high-advantage areas to the neighbouring regions, gradually reduce the regional differences of the main producing provinces, and promote the formation of modern apple producing areas with advantageous and distinctive characteristics.

The influencing factors of APCA are significantly heterogeneous. Hence, the role of influencing factors should be tailored and targeted to local conditions. Farmland infrastructure is an important guarantee for apple production, and the construction of farmland infrastructure should be emphasised and strengthened. Also, the construction of agricultural socialisation service system should be improved, and an effective market early warning mechanism should be established to give fruit farmers stable income expectations. Additionally, the government needs to focus on the organisation and cultivation development of large fruit farmers, co-operatives and family farms in the region, to help fruit farmers open up the market and improve comparative efficiency. For areas with high labour mobility, it is necessary to establish a professional farmer system and introduce relevant preferential policies to attract foreign farmers to contract orchard production and management.

## Supporting information

S1 Data The APCA, SAI and EAI data of the eight main producing provinces in China. (XLSX)
